# *MET* 14外显子跳跃突变NSCLC靶向治疗专家共识

**DOI:** 10.3779/j.issn.1009-3419.2023.102.19

**Published:** 2023-06-20

**Authors:** 中国老年保健协会肺癌专业委员会

**Keywords:** 肺肿瘤, MET 14外显子跳跃突变, MET抑制剂, Lung neoplasms, MET exon 14 skipping mutation, MET inhibitors

## Abstract

间质-上皮细胞转化因子（mesenchymal-epithelial transition factor, MET）14外显子跳跃突变主要由于c-Cbl酪氨酸结合位点丢失导致，从而引起蛋白酶体介导的MET蛋白降解率降低，引发下游通路的持续激活，最终导致肿瘤发生。非小细胞肺癌（non-small cell lung cancer, NSCLC）患者中MET 14外显子跳跃突变的发生率为0.9%-4.0%，晚期NSCLC患者应进行MET 14外显子跳跃突变的检测，以筛选MET抑制剂靶向治疗获益人群。MET抑制剂客观缓解率均较高，安全性良好，为MET 14外显子跳跃突变NSCLC患者带来新希望。中国老年保健协会肺癌专业委员会组织专家结合临床实践经验及循证医学证据，针对MET 14外显子跳跃突变NSCLC靶向治疗的临床问题给予专家建议，制定《MET 14外显子跳跃突变NSCLC靶向治疗专家共识》，旨在为中国医师的临床实践提供规范化指导。

## Abstract (en)

The mesenchymal-epithelial transition factor (MET) exon 14 skipping mutation is mainly caused by the loss of c-Cbl tyrosine binding site. This mutation could result in a decrease in the degradation rate of proteasome-mediated MET proteins, trigger continuous activation of downstream pathways, and ultimately lead to tumorigenesis. The incidence of MET exon 14 skipping mutation in patients with non-small cell lung cancer (NSCLC) is 0.9% to 4.0%. Patients with advanced NSCLC are recommended to test MET exon 14 skipping mutations who may benefit from MET inhibitors-targeted therapy. MET inhibitors have a high objective response rate and good safety profiles, which could prolong the survival of NSCLC patients with MET exon 14 skipping mutations. The Lung Cancer Specialty Committee of Chinese Elderly Health Care Association organized multidisciplinary experts to give suggestions on the important issues of clinical aspects for targeted therapy of MET exon 14 skipping mutation in NSCLC according to the clinical practice experiences and evidences based medicine. "Expert Consensus on Targeted Therapy of NSCLC with MET Exon 14 Skipping Mutation" is proposed, aiming to provide standardized guidances for the clinical practice of Chinese physicians.

非小细胞肺癌（non-small cell lung cancer, NSCLC）是肺癌中最常见的病理类型，由许多组织学特征和已知的特定驱动基因突变组成，大多数患者在确诊时已属晚期。近20年，靶向治疗给携带有表皮生长因子受体（epidermal growth factor receptor, EGFR）、间变性淋巴瘤激酶（anaplastic lymphoma kinase, ALK）等基因异常的NSCLC患者的治疗带来了极大的生存机会^[1,2]^。间质-上皮细胞转化因子（mesenchymal-epithelial transition factor, MET）基因是NSCLC的重要肿瘤驱动基因，被认为是继EGFR、ALK和c-ros原癌基因1酪氨酸激酶（c-ros oncogene 1 receptor tyrosine kinase, ROS1）之后的重要治疗靶点，针对MET基因突变的靶向药物受到越来越多的关注。MET基因异常包括MET 14外显子跳跃突变、基因扩增、基因融合和蛋白过表达。其中，针对MET 14外显子跳跃突变的靶向药物发展最快，需要对其临床应用进行规范与建议。

MET基因也称为c-MET，为原癌基因，位于人类第7号染色体长臂7q21-31，DNA长度约为125 kb，包含21个外显子和20个内含子。MET基因编码的MET蛋白，是肝细胞生长因子（hepatocyte growth factor, HGF）的酪氨酸激酶受体家族中的一员，其在胚胎发育和组织再生过程中均发挥着重要的作用^[3]^。HGF与MET的细胞外结构域结合，促使MET发生二聚化、酪氨酸磷酸化，激活众多下游信号通路，从而发挥促进细胞增殖、细胞生长、细胞迁移、侵袭及血管生成等效应，在正常组织发育和肿瘤进展中发挥着重要作用^[4]^。

肿瘤发生时，基质细胞通过自分泌的方式过度分泌HGF，肿瘤细胞的MET过表达来反馈基质细胞的异常活化，从而导致肿瘤细胞中MET的进一步紊乱^[5]^。MET 14外显子跳跃突变是MET降解失活的主要原因。MET 14外显子对应编码141个氨基酸，其所在的近膜结构域是c-MET的关键负性调控区，参与c-MET蛋白的泛素化和降解。MET 14外显子基因突变会引起MET 14外显子跳跃突变，使得产生的异常蛋白缺失了跨膜结构域，进而导致c-MET蛋白泛素化障碍、c-MET稳定性增加和降解率降低，引起下游信号的持续激活，最终成为肿瘤的驱动基因。

在NSCLC人群中，中国MET 14外显子跳跃突变的比例为0.9%-2.0%^[6⇓⇓-9]^，中国香港地区和中国台湾地区分别为2.6%^[10]^和3.3%^[11]^，略低于国外人群数据（2%-4%）^[12]^。其中，MET 14外显子跳跃突变通常发生在老年患者中^[13]^，中位年龄72.5岁^[14]^。MET 14外显子跳跃突变在肺腺癌患者中的发生率约3%^[12]^，在肺鳞癌中为1%-2%^[15,16]^。有研究^[13,17]^发现，13%-22%的肺肉瘤样癌患者会发生MET 14外显子跳跃突变。研究^[18]^显示，携带MET 14外显子跳跃突变的NSCLC患者有0.3%-10.0%同时携带EGFR突变，6.4%-28.5%同时携带EGFR基因扩增。MET 14外显子跳跃突变通常与高侵袭性、抗肿瘤治疗的耐药性和不良预后相关^[10,19]^。多项临床研究^[20,21]^显示，MET抑制剂在MET 14外显子跳跃突变晚期NSCLC患者中展示了良好的抗肿瘤活性以及安全性。

本共识专家组成员来自全国多个省市自治区，包括胸外科、肿瘤内科及呼吸内科等多学科专家。专家组成员讨论汇总MET 14外显子跳跃突变NSCLC靶向治疗临床实践中面临的重要问题，并梳理已发表的临床研究证据，进行全面的文献检索，基于循证医学证据并结合自身临床经验对临床问题进行充分讨论，对于关键的问题进行投票，汇总专家意见并统计投票结果，最终形成本共识的推荐内容。执笔专家整理出共识初稿后，发送给其他专家组成员进行审核修改，完成最终定稿。本共识基于现有循证医学证据以及专家组广泛认可的临床经验提出推荐内容。

检索PubMed、EMBASE数据库，检索截止时间为2023年2月1日，检索关键词包括：非小细胞肺癌、c-MET、MET 14外显子、MET 14外显子跳跃突变；英文关键词包括Non-Small Cell Lung Cancer、c-MET、MET exon 14、MET exon 14 skipping mutation。检索的文献限于系统综述、荟萃分析和随机对照临床研究，剔除重复文献、述评、编辑点评、来信、新闻报道、叙述性综述以及后续未发表于同行评审期刊的会议摘要。

**共识一致性级别：**

**（1）投票100%一致：所有专家完全达成共识，一致推荐；（2）投票75%-99%一致：绝大多数专家达成共识，推荐；（3）投票50%-74%一致：多数专家达成共识，少数专家存在分歧但推荐；（4）投票<50%一致：不推荐。**

**问题1：哪些NSCLC患者需要进行MET 14外显子跳跃突变检测?**

**共识推荐1：推荐所有晚期NSCLC（包括腺癌、鳞癌、肉瘤样癌等）患者在诊断时常规进行MET 14外显子跳跃突变检测（共识一致性级别：一致推荐）。**

2014年，美国国立综合癌症网络（National Comprehensive Cancer Network, NCCN）NSCLC指南扩展了包括MET扩增和突变在内的生物标志物，并鼓励广泛开展基因检测，指导罕见靶点用药和临床试验的参与。2021年，我国发布《非小细胞肺癌分子病理检测临床实践指南（2021版）》^[22]^，将MET 14外显子跳跃突变列为必检。《中国临床肿瘤学会（CSCO）非小细胞肺癌诊疗指南2022》^[23]^推荐对于不可手术III期及IV期非鳞癌患者进行MET 14外显子跳跃突变的检测，且推荐等级上调至I级推荐。2023年NCCN指南建议非鳞NSCLC患者均进行MET 14外显子跳跃突变检测，晚期鳞癌患者可考虑进行此项检测。

**问题2：哪些检测方法可用于MET 14外显子跳跃突变?**

**共识推荐2：可使用实时荧光定量逆转录聚合酶链反应（reverse transcription quantitative-polymerase chain reaction, RT-qPCR）、基于DNA的下一代测序（next-generation sequencing, NGS）或RNA-NGS对MET 14外显子跳跃突变进行检测，必要时可相互验证或补充检测（共识一致性级别：一致推荐）。**

MET 14外显子跳跃突变的检测方法主要包括RT-qPCR、DNA-NGS或RNA-NGS^[24]^，不同检测方法各有其优缺点，必要时可相互验证或补充检测。

RT-qPCR能够较准确快速地探测到MET 14外显子跳跃突变的存在，兼具敏感性和特异性，检测周期短，适用于组织及细胞学样本^[25]^。DNA-NGS可在DNA层面直接鉴定出MET 14外显子区域碱基对突变的具体情况，是目前检测MET 14外显子跳跃突变最常用的手段，但检出率会受到引物设计或探针覆盖及生物信息分析能力的影响，且周期相对较长，优选肿瘤组织或细胞学样本，当上述样本无法获得时，也可考虑液体活检样本。RNA-NGS在RNA水平上检测MET 13/15外显子融合来判断是否发生MET 14外显子跳跃突变，即使肿瘤细胞数量较少的样本，也可保持较高敏感性。

《非小细胞肺癌MET临床检测中国专家共识》^[24]^指出晚期NSCLC MET 14外显子跳跃突变检测首选肿瘤组织/细胞学样本。组织样本包括手术样本和活检样本，细胞学样本包括胸/腹腔积液、支气管刷检、支气管内超声引导细针穿刺活检样本、痰/肺泡灌洗液等，评估满足检测要求后进行检测。对于不能获得组织或细胞学样本的晚期NSCLC患者，可考虑循环肿瘤细胞（circulating tumor cell, CTC）检测。当出现阴性结果时，应注明假阴性的可能性，必要时建议重新取样后复测^[24,26]^。DNA-NGS检测结果阴性时，可考虑采用RNA样本补充检测。

**问题3：MET 14外显子跳跃突变的NSCLC患者治疗方案如何选择?**

**共识推荐3：对于局部晚期及转移性MET 14外显子跳跃突变的NSCLC患者，目前的临床数据提示MET抑制剂的疗效良好，可以优先考虑采用高选择性Ib类MET酪氨酸激酶抑制剂（tyrosine kinase inhibitors, TKIs）赛沃替尼（Savolitinib）、特泊替尼（Tepotinib）、卡马替尼（Capmatinib）、伯瑞替尼（Bozitinib）、谷美替尼（Glumetinib）等进行治疗。另外，在等待分子检测结果的同时，患者可接受标准系统治疗。同时鼓励患者参与临床研究，进行更好的疾病管理（共识一致性级别：一致推荐）。**

MET 14外显子跳跃突变NSCLC患者一线接受化疗的整体疗效不佳，既往研究^[11]^显示，中位总生存期（overall survival, OS）仅为6.7个月。免疫治疗疗效同样也不理想，MET 14外显子跳跃突变局部晚期或转移性NSCLC患者一线应用免疫治疗的客观缓解率（objective response rate, ORR）为17%-35.7%，中位无进展生存期（progression-free survival, PFS）仅1.9个月-4.9个月^[27,28]^。对于初诊局部晚期或转移性NSCLC患者，在基因检测结果明确之前，如患者病情需要，可接受以化疗为基础的标准系统性治疗，根据患者具体情况选择相应的用药方案。如果后续基因检测结果明确存在MET 14外显子跳跃突变，可选择继续完成系统治疗，或者停止系统治疗，改为MET小分子TKIs治疗。

作用于MET 14外显子跳跃突变的靶向药物主要有单克隆抗体和TKIs。游离的c-MET可采用独特的自抑制构象，其激活环可通过D1228和K1110之间的盐桥，锁定在ATP三磷酸结合位点上。根据结构组合的不同，MET-TKIs可分为3种类型（I类、II类、III类）。根据结合点位的不同，又可分为单靶点和多靶点两类，单靶点MET抑制剂也叫高选择性MET抑制剂，能够锚定MET较为独特的铰链区域，选择性抑制MET激酶，如赛沃替尼、特泊替尼、伯瑞替尼、谷美替尼；多靶点MET抑制剂[如克唑替尼（Crizotinib）]能够靶向作用于多个激酶，其血管内皮生长因子受体（vascular endothelial growth factor receptor, VEGFR）活性远高于MET活性，用于靶向MET的疗效不尽理想。

MET 14外显子跳跃突变的口服靶向药物在局部晚期或转移性NSCLC中的关键临床研究数据^[20,21,29⇓-31]^见表1。

**表1:** 治疗MET 14外显子跳跃突变的口服靶向药物在局部晚期或转移性NSCLC中关键临床研究数据

药物名称	治疗 线数	入组 例数	ORR （95%CI）	DCR （95%CI）	mDoR（月）（95%CI）	mPFS（月）（95%CI）	主要不良反应 （发生率≥20%）
克唑替尼^[29]^	二线及以上	69*	32% （21%-45%）	-	9.1（6.4-12.7）	7.3 （5.4-9.1）	水肿、视觉障碍、恶心、腹泻、呕吐
卡马替尼^[21]^	一线	60	68.3% （55%-79.7%）	98.3%（91.1%-100%）	16.6（8.4-22.1）	12.5（8.3-18.0）	外周水肿、恶心、血肌酐升高、呕吐、呼吸困难、疲乏、食欲减退
	二线	31	48.4% （30.2%-66.9%）	90.3%（74.2%-98.0%）	6.93（4.17-NE）	8.11（4.17-9.86）	
	二线及以上	69	41% （29%-53%）	78% （67%-87%）	9.7（5.6-13.0）	5.42（4.2-7.0）	
特泊替尼^[30]^	一线	99	46.5% （36.4%-56.8%）	65.7%（55.4%-74.9%）	11.1 （7.2-NE）	8.5（6.7-11.0）	外周水肿、恶心、腹泻
赛沃替尼^[20]^	一线	70	49.2% （36.1%-62.3%）	93.4% （84.1%-98.2%）	8.3 （5.3-16.6）	6.9 （4.6-8.3）	外周水肿、恶心、低白蛋白血症、转氨酶升高、呕吐、食欲减退、乏力、低血钾、贫血、咳嗽
谷美替尼^[31]^	一线	44	71% （55%-83%）	89% （74%-91%）	15.0（6.3-NE）	11.7（7.6-21.9）	外周水肿、头痛、恶心、食欲减退、低白蛋白血症、ALT升高、呕吐
	二线及以上	35	60% （42%-76%）	77%（60%-90%）	8.2 （5.1-NE）	7.6 （4.1-9.6）	
	总人群	79	66% （54%-76%）	84% （74%-91%）	8.3 （6.2-NE）	8.5 （7.6-9.7）	
伯瑞替尼	一线	52	75% （61.1%-86.0%）	96.2%	16.7	12	外周水肿、低蛋白血症、转氨酶升高

*纳入患者不仅仅为MET 14外显子跳跃突变，包含其他MET改变。 MET：间质-上皮细胞转化因子；NSCLC：非小细胞肺癌；NE：未评估；ORR：客观缓解率；DCR：疾病控制率；DoR：缓解持续时间；PFS：无进展生存期；ALT：谷丙转氨酶。

**非选择性多靶点MET抑制剂** 最初进入临床探索的MET-TKIs为非选择性多靶点MET抑制剂，如克唑替尼和卡博替尼。

探索克唑替尼用于二线及以上MET 14外显子改变局部晚期或转移性NSCLC后线治疗的临床研究^[29,32]^纳入69例患者，结果显示ORR为32%，中位缓解持续时间（duration of response, DoR）为9.1个月，中位PFS为7.3个月，初步显示了MET-TKIs的疗效。在另外3项克唑替尼治疗MET 14外显子突变NSCLC的II期研究^[33⇓-35]^中，分别入组了52例、25例和28例患者，ORR分别为27%、25%和18%，中位PFS分别为4.4个月、3.6个月和3.5个月。一项来自美国的MET抑制剂治疗MET 14外显子突变NSCLC的多中心回顾性研究^[36]^共纳入148例患者，其中有22例接受克唑替尼治疗，中位PFS达7.4个月。我国的一项回顾性分析^[37]^从11,306例中国患者中筛选出125例MET 14外显子突变的NSCLC患者，其中44例患者接受了克唑替尼一线治疗，其PFS显著长于接受化疗的患者（8.5个月 vs 4.0个月，P=0.041），但两组患者的OS相似（11.3个月 vs 12.0个月，P=0.66）。

卡博替尼是口服的多靶点TKIs，可以抑制c-MET、VEGFR2、KIT、TIE3、FLT3和RET活性。目前有研究在进一步探索卡博替尼联合厄洛替尼治疗既往治疗失败的EGFR突变阴性且伴有MET活化（过表达、扩增或者突变）的NSCLC的有效性（NCT01639508）。福瑞替尼（Foretinib）也是多靶点小分子TKIs，可以靶向MET、RON、AXL、TIE-2、VEGFR和ROS-1，目前对于NSCLC的治疗仍在探索阶段。格来替尼是一个靶向MET和AXL的小分子TKIs，其与非活性的MET构象结合能够抑制与I型MET抑制剂耐药相关的MET环突变活化。初步研究^[38]^显示格来替尼在MET 14外显子跳跃突变体和MET扩增的模型中的抗肿瘤作用。在一项格来替尼治疗伴有MET/AXL基因改变的进展期/不可手术NSCLC和其他实体瘤的I期研究^[39]^中，格来替尼最大耐受剂量分别为1,050 mg bid（胶囊）和750 mg bid（片剂），对于MET/AXL突变或扩增的NSCLC，ORR为25.9%，其中对于MET突变人群ORR达30%，共有6例获得部分缓解（partial response, PR）的患者伴有MET 14外显子跳跃突变。同时还有一项探索格来替尼联合纳武利尤单抗（Nivolumab）治疗NSCLC疗效和安全性的II期研究已经完成（NCT02954991），研究结果尚未公布。S49076是一个抑制MET、AXL/MER和成纤维细胞生长因子受体（fibroblast growth factor receptor, FGFR）的多靶点TKIs。在一项S49076联合吉非替尼治疗EGFR-TKIs治疗失败的伴有MET和/或AXL基因异常的NSCLC的I期研究^[40]^中，共14例患者入组，S49076推荐剂量600 mg qd，最佳疗效是2例达到PR（1例患者MET基因异常，1例患者MET和AXL基因均异常），8例患者达到疾病稳定（stable disease, SD）。同时S49076与放疗联合治疗NSCLC的相关研究^[41]^也在进行中。

**高选择性单靶点MET抑制剂** 目前，多个高选择性、高特异性的MET抑制剂，包括卡马替尼、赛沃替尼、特泊替尼、谷美替尼和伯瑞替尼等，正在逐步开发和应用中，这些药物特异性地作用于MET 14外显子跳跃突变，显示出更为优越的疗效。

卡马替尼作为首个美国食品药品监督管理局（Food and Drug Administration, FDA）获批的MET抑制剂，是一种高选择性的IB型MET-TKIs。GEOMETRY mono-1研究^[21]^是一项前瞻性、非随机、开放标签的II期研究，探索了卡马替尼用于MET 14外显子跳跃突变的晚期NSCLC患者的疗效和安全性。2021年ASCO更新GEOMETRY mono-1研究数据^[42]^，该项研究共纳入160例存在MET 14外显子跳跃突变的NSCLC患者，结果显示，在60例初治患者中盲态独立评审委员会（Blind Independent Review Committee, BIRC）评估的ORR为68.3%，DCR为98.3%，中位PFS为12.5个月，中位OS达25.5个月；该研究共入组100例MET 14外显子跳跃突变经治患者，其中卡马替尼二线治疗的ORR为51.6%，DCR为90.3%，二线及后线治疗的ORR为40.6%，DCR为78.3%。与接受后线治疗的患者相比，接受一线治疗的患者具有更高的ORR、DCR和PFS。最常见的不良反应是外周水肿和恶心，大多为1级或2级，且不良反应可管可控。基于GEOMETRY mono-1研究，卡马替尼于2020年5月6日被美国FDA批准用于治疗具有MET 14外显子跳跃突变的转移性NSCLC成人患者。

特泊替尼是一种口服、高选择性的IB型MET-TKIs。VISION研究^[30]^为一项探索特泊替尼用于MET 14外显子跳跃突变的转移性NSCLC疗效和安全性的II期临床研究。研究共纳入152例含有MET 14外显子跳跃突变的NSCLC患者，均接受特泊替尼治疗，其中99例患者至少随访9个月。结果显示，99例患者整体ORR为46%，其中液体活检组（从血清中检测出MET 14外显子跳跃突变）患者ORR为48%，组织活检组（从新鲜或石蜡肿瘤组织中检测出MET 14外显子跳跃突变）患者ORR为50%，联合活检组患者ORR为46%，全组疾病控制率（disease control rate, DCR）为65.7%，中位DoR为11.1个月，中位PFS为8.5个月。常见的不良反应包括外周水肿、恶心、腹泻、血肌酐升高和低蛋白血症。2022年世界肺癌大会（World Conference on Lung Cancer, WCLC）口头报告了特泊替尼II期VISION研究验证性队列的初步研究结果^[43]^。结果显示，全组患者ORR为54.7%，中位DoR为20.8个月，中位PFS为13.8个月，中位OS为18.8个月。其中，经过组织检测确认存在MET 14外显子跳跃突变的初治患者中，ORR为62.3%，中位DoR未达到，中位PFS为15.9个月，中位OS为22.7个月，结果提示，特泊替尼用于MET 14外显子跳跃突变的NSCLC患者具有良好的疗效。2020年3月，特泊替尼在日本获批用于治疗MET 14外显子跳跃突变的晚期NSCLC患者。2021年2月3日，FDA批准特泊替尼用于MET 14外显子跳跃突变的晚期NSCLC患者。2022年3月30日，中国国家药品监督管理局（National Medical Products Administration, NMPA）药品审评中心（Center for Drug Evaluation, CDE）已受理特泊替尼用于治疗MET 14外显子跳跃突变局部晚期或转移性的NSCLC新药上市申请。

赛沃替尼是一种高选择性的IB型MET-TKIs，一项单臂II期临床试验^[20]^探索了赛沃替尼用于MET 14外显子跳跃突变的NSCLC患者的疗效和安全性。研究纳入70例MET 14外显子跳跃突变的NSCLC患者接受赛沃替尼治疗。结果显示，疗效可评估集ORR为49.2%，DCR为93.4%，中位PFS为6.9个月。发生率超过20%的不良反应为外周水肿、恶心、谷草转氨酶（aspartate transaminase, AST）/谷丙转氨酶（alanine transaminase, ALT）升高、呕吐和低蛋白血症，≥3级的不良反应发生率为41.4%，导致终止治疗的不良反应发生率为14.3%。赛沃替尼长期疗效和安全性数据的亚组分析^[44]^结果进一步证实了赛沃替尼对MET 14外显子跳跃突变NSCLC患者具有良好的疗效和安全性。2021年6月23日赛沃替尼被NMPA批准用于含铂化疗后疾病进展或不耐受标准含铂化疗、具有MET 14外显子跳跃突变的局部晚期或转移性NSCLC成人患者。目前赛沃替尼是我国唯一进入医保的高选择性MET-TKIs。

谷美替尼是一种结构全新的IB型高选择性MET-TKIs，其关键II期GLORY临床研究^[31]^探索了在既往接受不超过2种全身治疗或未接受全身治疗的MET 14外显子跳跃突变局部晚期或转移性NSCLC患者中的疗效和安全性。研究共纳入79例患者，包括44例初治及35例经治患者。BIRC评估的初治患者ORR为71%，DCR为89%，中位DoR为15个月，中位PFS为11.7个月，中位OS未达到；经治患者ORR为60%，DCR为77%，中位DoR为8.2个月，中位PFS为7.6个月，中位OS为16.2个月。总人群的ORR为66%，DCR为84%，中位DoR为8.3个月，中位PFS为8.5个月，中位OS为17.3个月。14例基线有脑转移的患者中有13例被纳入疗效分析集，经BIRC评估（未选择脑转移瘤作为靶病灶）的ORR为85%，研究者将5例患者的脑转移瘤作为靶病灶，均观察到颅内客观缓解。谷美替尼的总体安全耐受性良好，主要的不良反应包括水肿、头痛、恶心、食欲减退、低白蛋白血症、ALT升高、呕吐等。2023年3月7日，NMPA正式批准化药1类新药海益坦®谷美替尼片（研发代号：SCC244）上市，用于治疗MET 14外显子跳跃突变的局部晚期或转移性NSCLC成人患者。

伯瑞替尼为一种高选择性IB型MET-TKIs，其I期研究纳入37例MET异常的经治但未接受过c-MET抑制剂靶向治疗的局部晚期或转移性晚期NSCLC患者，均接受伯瑞替尼治疗，其中11例患者仅携带MET 14外显子跳跃突变。结果显示，在携带MET 14外显子跳跃突变的患者中，ORR为66.7%，安全性可控^[45]^。最新II期关键注册研究结果显示，接受伯瑞替尼治疗的52例晚期MET 14外显子跳跃突变NSCLC患者已达到预设主要终点。由独立评审委员会（Independent Review Committee, IRC）判定的ORR达到75%（95%CI: 61.1%-86.0%），DoR达到16.7个月，PFS达到12个月。初治患者ORR达到77.1%，标准治疗失败患者ORR达到70.6%，且对颅内转移灶有效，颅内ORR达到62.5%，可测量中枢神经系统（central nervous system, CNS）颅内ORR达到100%（未发表数据）。基于此研究，伯瑞替尼目前已被CDE接受上市申报受理并拟纳入优先评审。

其他药物除了MET-TKIs外，以MET为靶点的双特异性抗体和抗体偶联药物（antibody-drug conjugate, ADC）近年来也取得了一定的突破。埃万妥单抗（Amivantamab）是一款EGFR/c-Met靶向双特异性抗体，于2021年5月被FDA批准用于治疗接受含铂化疗失败后病情进展、EGFR基因20外显子插入突变阳性的转移性NSCLC成人患者，CHRYSALIS研究^[46]^探索了埃万妥单抗在MET 14外显子跳跃突变NSCLC患者中的疗效和安全性，其初步结果在2022年美国临床肿瘤学会（American Society of Clinical Oncology, ASCO）大会上发表。研究共纳入55例患者，可评估患者46例，其中7例患者既往未接受过治疗，15例患者既往接受过除MET-TKIs外的治疗，24例接受过MET-TKIs治疗。结果显示，全组ORR为33%，初治患者ORR为57%，经治但既往未接受过MET-TKIs患者的ORR为47%，接受过MET-TKIs患者的ORR为17%，安全性整体可控。维汀-特立妥珠单抗（Telisotuzumab Vedotin）是一类靶向c-Met的ADC，其II期临床研究LUMINOSITY研究^[47]^探索了维汀-特立妥珠单抗作为二线或三线治疗用于c-MET过表达NSCLC患者的疗效和安全性。该研究在2022年ASCO大会上公布了其中期分析结果，截至报告时，共纳入136例患者，初步结果显示，在EGFR野生型非鳞NSCLC患者中，c-Met表达高水平组ORR为52.2%，c-Met表达中等水平组ORR为24.1%，主要不良反应为外周神经感觉异常、恶心和低白蛋白血症。维汀-特立妥珠单抗于2022年1月被FDA授予突破性疗法资格，用于治疗在铂类治疗期间或之后进展的局部晚期或转移性EGFR野生型、c-Met过表达非鳞NSCLC患者。目前还有多种在研的以MET为靶点的MET-TKIs、双特异性抗体和ADC药物，未来或能为MET 14外显子跳跃突变NSCLC患者提供更多治疗选择。

**问题4：对于程序性死亡配体1（programmed cell death ligand 1, PD-L1）阳性的MET 14外显子跳跃突变NSCLC患者治疗方案如何选择?**

**共识推荐4：MET 14外显子跳跃突变NSCLC PD-L1阳性（≥1%）及高表达（≥50%）比例高，但通常肿瘤突变负荷（tumor mutation burden, TMB）低，免疫单药治疗效果差，特别是病理类型为肉瘤样癌（共识一致性级别：一致推荐）。**

PD-L1在MET 14外显子跳跃突变的NSCLC患者中倾向于高水平表达。Xu等^[8]^对13例PD-L1阳性的MET 14外显子跳跃突变NSCLC患者进行检测，其中9例高表达，占比69.2%。Sabari等^[27]^共纳入147例MET 14外显子跳跃突变的肺癌患者，在其中可评估的111例患者样本中PD-L1≥1%为63%，PD-L1≥50%为41%，病理类型最常见的是腺癌，其次为肉瘤样癌。2022年WCLC大会上，一项研究^[48]^对比了138例MET 14外显子跳跃突变和5,162例MET野生型NSCLC患者的PD-L1表达特征，可见MET 14外显子跳跃突变患者的PD-L1高表达比例显著更高（56.7% vs 29.8%），但TMB高（TMB-H，定义为TMB≥10 mt/Mb）的患者显著更少（16.3% vs 48.5%）。

2023年ASCO大会报道了一项真实世界研究^[49]^，对比分析了真实世界临床实践中MET 14外显子跳跃突变NSCLC患者一线接受卡马替尼、免疫治疗或化疗的疗效对比。共纳入了287例明确MET 14外显子跳跃突变的晚期NSCLC患者，中位年龄63.3岁，71%为男性。分析发现，MET 14外显子跳跃突变的患者一线接受卡马替尼治疗相较于含铂化疗、免疫单药或免疫联合化疗具有更高的真实世界ORR（real world ORR, rwORR）及更长的真实世界PFS（real world PFS, rwPFS）和OS数据，rwORR为73.4%，18个月的rwPFS为68%，18个月的OS为92.6%。2022年欧洲肿瘤内科学会（European Society for Medical Oncology, ESMO）大会一项真实世界研究^[50]^，根据PD-L1表达水平分析美国晚期MET 14外显子跳跃突变NSCLC患者的基线特征、治疗模式和临床结局。共纳入138例晚期MET 14外显子跳跃突变NSCLC患者，其中41.3%的患者PD-L1肿瘤细胞阳性比例（tumor cell proportion score, TPS）高表达（TPS≥50%）、13.7%的PD-L1低表达（TPS 1%-49%）、6.5%的PD-L1无表达（TPS<1%）、38.4%的患者未检测出PD-L1表达情况。在治疗方案选择上，PD-L1高表达组最常见一线方案为免疫单药治疗，PD-L1低表达组为免疫联合化疗，PD-L1阴性组为化疗。但即使PD-L1高表达，免疫治疗对MET 14外显子跳跃突变NSCLC患者疗效仍欠佳，免疫治疗的中位PFS和中位OS低于其他一线方案（中位PFS：4.1个月 vs 5.4个月；中位OS：11.4个月 vs 17.0个月），而选择性MET-TKIs在MET 14外显子跳跃突变NSCLC患者一线治疗中的应用比例有限，在二线治疗中接受选择性MET-TKIs的比例高于其他治疗方案，未报道PD-L1高表达患者一线应用MET抑制剂治疗的PFS和OS数据。

在GEOMETRY mono-1研究中，MET 14外显子跳跃突变初治患者（队列5b和队列7，n=60）接受卡马替尼治疗。队列7中32例初治患者接受卡马替尼治疗，ORR为68.8%（95%CI: 50.0%-83.9%），中位DoR为16.59个月（95%CI: 50.0-83.9），中位PFS达12.45个月（95%CI: 6.87-20.50）^[51]^；如果进行非头对头比较，靶向治疗的PFS和OS在数值上具有显著优势。需有待更大样本量的研究进一步探索PD-L1阳性及高表达的MET 14外显子的NSCLC患者的最佳一线治疗方案。

**问题5：对于MET 14外显子跳跃突变合并共突变的NSCLC患者治疗方案如何选择?**

**共识推荐5：MET 14外显子跳跃突变通常与NSCLC其他已知驱动基因改变不共存；但合并共突变者，对靶向治疗药物疗效及患者预后有一定潜在影响；合并TP53突变率与EGFR阳性、ALK阳性NSCLC相似（共识一致性级别：一致推荐）。**

在MET 14外显子跳跃突变共存的基因突变中最常见的是TP53突变。2022年WCLC大会一项研究^[52]^检测935份NSCLC患者肿瘤样本，其中有37.5%的MET 14外显子跳跃突变NSCLC检测到TP53共突变，与EGFR突变和ALK融合的NSCLC中的TP53共突变的比例（46.2%, 21.2%）相似^[8]^。除TP53突变外，有13.6%的NSCLC患者发生POT1、TERT和KRAS基因突变。MET 14外显子跳跃突变样本中同时发生KRAS突变、EGFR突变的比例分别为3.2%和0.65%^[53]^。

在MET 14外显子跳跃突变的肿瘤组织中同时检测到TP53和POT1突变可能会对赛沃替尼治疗结果产生不利影响，但因可分析患者数量较少，共突变对赛沃替尼治疗结果的相关性必须在更大的队列研究中得到证实^[54]^。MET-TKIs改善患者预后的价值，仍有待更多更广泛的研究加以评估。

**问题6：MET抑制剂能否使MET 14外显子跳跃突变的NSCLC患者在围手术期治疗中获益?**

**共识推荐6：MET抑制剂在MET 14外显子跳跃突变的早期NSCLC患者的围手术期应用，尚缺乏高级别证据支持；基于既往个案报道，对于高龄、拒绝化疗或存在化疗禁忌证的患者可进行探索性应用；建议针对MET 14外显子跳跃突变的早期NSCLC患者开展多中心新辅助和辅助治疗的临床试验（共识一致性级别：一致推荐）。**

目前，关于MET抑制剂在MET 14外显子跳跃突变的早期NSCLC患者的围手术期应用，缺乏大规模循证医学证据，仅有一些个案报道。在Rotow等^[55]^的一项个案报道中，1例右上肺肿物直径达15 cm并纵隔淋巴结肿大、具有MET 14外显子跳跃突变及MET扩增的亚裔女性患者，经过8个月克唑替尼治疗后，手术治疗后病理确认为完全缓解，并于术后1周后继续克唑替尼辅助治疗。全程药物治疗耐受良好，至术后6个月个案发表时仍未复发。另有4篇有关MET抑制剂术前应用新辅助治疗的个案^[56⇓⇓-59]^，报道了患者在手术前应用4周-10周赛沃替尼后缩瘤或降期，随后进行根治性手术。关于后续的术后辅助治疗，这些案例中包括继续给予赛沃替尼辅助治疗^[56,57]^、降期后按照术后分期未予辅助治疗而持续随访观察^[58]^、术后进行标准培美曲塞加卡铂的辅助化疗^[59]^。当前，有一项关于卡马替尼在具有MET 14外显子跳跃突变或扩增的NSCLC围手术期应用的II期临床试验（NCT04926831, Geometry-N）正在进行中。由于尚缺乏MET抑制剂围手术期应用的高级别证据，因此建议针对MET 14外显子跳跃突变的早期NSCLC患者，开展更多的多中心新辅助和辅助治疗的临床试验，探索MET抑制剂在围手术期治疗中的安全性和疗效。

**问题7：MET-TKIs不良反应及管理**

**共识推荐7：MET-TKIs常见共同不良反应包括外周水肿、恶心呕吐、肝脏毒性。MET 14外显子跳跃突变NSCLC患者是一个年龄更大的人群，需要更加重视不良反应管理（共识一致性级别：一致推荐）。**

MET-TKIs最常见的不良反应为外周水肿，总体发生率为50%-63%。其次为恶心呕吐，发生率为26%-46%。3级以上治疗相关不良反应（treatment related adverse events, TRAEs）为AST升高（2%-13%）、ALT升高（3%-10%）及周围水肿（1%-11%）。TRAEs导致减量和停药的比例分别为33%-38%和7%-14%。MET-TKIs不良反应汇总见附表1。

**附表1:** MET-TKIs不良反应汇总

不良反应	克唑替尼			特泊替尼			卡马替尼			塞沃替尼			伯瑞替尼			谷美替尼	
	1级-4级	≥3级		1级-4级	≥3级		1级-4级	≥3级		1级-4级	≥3级		1级-4级	≥3级		1级-4级	≥3级
水肿	51%	1%		63%	7%		50%	11%		54%	9%		55.2%	6.7%		59.3%	11.3%
肝功能损害	17%	4%		7%	5%		11%	7%		39%	13%		8.1%	4.8%			
血胆红素升高																20.3%	0.3%
ALT升高																17.3%	1.0%
AST升高																13.3%	1.3%
恶心呕吐	41%	0%		26%	1%		36%	1%		46%	0%		17.6%	0%		54%	4.0%
腹泻	39%	0%		22%	1%		9%	0%		NR	NR		5.7%	0%		NR	NR
肌酐升高	NR	NR		18%	1%		19%	0%		NR	NR		10%	0%		9.0%	0%
低白蛋白血症	NR	NR		16%	2%		NR	NR		23%	0%		19.0%	1.0%		28.7%	0.7%
淀粉酶升高	NR	NR		11%	3%		9%	7%		NR	NR		7.6%			NR	NR
食欲减退	19%	0%		8%	1%		13%	1%		20%	0%		6.2%	0%		33%	2.0%
疲劳	23%	0%		7%	1%		13%	3%		NR	NR		12.9%	0%		19.7%	2.3%
视觉障碍	45%	0%		NR	NR		NR	NR		NR	NR		NR	NR		NR	NR
便秘	20%	0%		NR	NR		NR	NR		NR	NR		NR	NR		7.0%	0%

MET-TKIs: 间质-上皮转化因子酪氨酸激酶抑制剂；ALT：谷丙转氨酶；AST：谷草转氨酶；NR：未达到。

大多数不良反应临床可控，无需减量和停药。作为临床医生，要了解MET-TKIs TRAEs严重程度、发生时间、药物减量和停止策略，还需要了解更多不良反应干预和管理策略的相关数据。MET-TKIs不良反应减量及停止策略见附表2、附表3。

**附表2:** MET-TKIs不良反应应对策略

不良反应严重程度	剂量调整
外周水肿	
1级-2级（体积或周长差异最大处比较，肢体间差异1级为 5%-10%，2级为10%-30%）	不需调整剂量
3级（体积或周长差异最大处比较，为30%以上）	暂停用药，直至恢复至1级：如可恢复至1级，则下调剂量后继续使用，否则永久停药
肝脏毒性	
ALT、AST或总胆红素异常	首先参考药品说明书中肝功能异常的建议或参考2019年欧洲肝脏研究学会指南和/或进行院内多学科诊疗会诊确定剂量调整方案
恶心呕吐	
1级-2级	不需调整剂量
3级	暂停用药，直至恢复至1级：如可恢复至1级，则下调剂量后继续使用，否则永久停药
肾功能损害	
轻度（肌酐清除率：60 mL/min-89 mL/min）和中度（肌酐清除率：30 mL/min-59 mL/min）肾损害	不需调整剂量
无需透析的严重肾损伤（肌酐清除率<30 mL/min）	减量谨慎服用，严密监测肾功能
间质性肺炎	
任何级别的药物相关间质性肺疾病/非感染性肺炎	永久停药
严重视力丧失	在评估严重视力丧失期间，停用本药

**附表3:** MET-TKIs减量方案

药物名称	减量方案
克唑替尼	起始剂量为250 mg bid，第一次减量至200 mg bid，第二次减量至250 mg qd，如果仍无法耐受250 mg qd，则永久停药
特泊替尼	起始剂量为450 mg qd，因不良反应需要减量，可减量至250 mg qd，如果仍无法耐受250 mg qd，则永久停药
赛沃替尼	体重≥50 kg，起始剂量为600 mg qd，第一次减量至400 mg qd，第二次减量至300 mg qd，第三次减量至200 mg qd； 体重<50 kg，起始剂量为400 mg qd，第一次减量至300 mg qd，第二次减量至200 mg qd
伯瑞替尼	起始剂量为200 mg bid，第一次减量至150 mg bid，第二次减量至100 mg bid
谷美替尼	起始剂量为300 mg qd，第一次减量至250 mg qd，第二次减量至200 mg qd，第三次减量至150 mg qd

外周水肿是各种MET-TKIs较为常见的不良反应，总体发生率为50%-63%，3级以上发生率为1%-11%。从开始服药到水肿发生的中位时间为50 d^[20]^。药物诱导的外周水肿被低估和误诊，经常导致处方联级。其发生主要涉及四种主要机制：毛细血管前小动脉血管舒张（血管扩张性水肿）、钠/水潴留（肾水肿）、淋巴不足（淋巴水肿）和毛细血管通透性增加（渗透性水肿）。外周水肿主要表现为肢体浮肿。接受MET-TKIs治疗的患者，可通过体重测量和相关临床检查进行监测。依据常见不良反应术语评定标准（Common Terminology Criteria for Adverse Events, CTCAE）v5.0，外周水肿的严重程度可分为三级^[60]^：在体积或周长差异最大处比较，一级肢体间差异为5%-10%，二级为10%-30%，三级则为30%以上。此外，二级外周水肿仅影响包括使用电话、烹调食物、家务在内的工具性日常生活活动（instrumental activities of daily living, IADL），而如果出现三级外周水肿则会影响到患者的个人基本日常生活活动能力（activities of daily living, ADL），包括进食、穿衣、洗澡等。

应对策略：对于外周水肿的管理流程，首先需明确病因，排除非药物因素导致的外周水肿，包括但不限于心脏疾病（通过脑钠肽及心脏超声检测）、肾脏疾病（血肌酐、尿常规）、肝脏疾病（肝酶、白蛋白）及深静脉血栓（D-二聚体检测、多普勒超声、磁共振静脉造影）。对于上述非药物因素需采取对应措施，如抗凝治疗、溶栓治疗等^[61]^。其次可通过限制食盐摄取、适当锻炼、弹力袜等方式控制水肿，必要时使用利尿剂进行治疗。1级-2级外周水肿，可继续使用MET-TKIs；3级则需暂停用药，直至恢复至1级：如可恢复至1级，则下调剂量后继续使用MET-TKIs，否则需永久停药^[62]^。

**问题8：如何克服MET-TKIs耐药?**

**共识推荐8：MET-TKIs的耐药不可避免，需通过精准检测明确MET-TKIs的耐药机制，针对不同耐药机制，可通过不同类型MET-TKIs之间的互换、采用系统性的化疗或化疗联合免疫治疗或抗血管治疗等多种方式克服耐药（共识一致性级别：一致推荐）。**

数个MET-TKIs获批用于治疗MET 14外显子跳跃突变晚期NSCLC，但靶向治疗后不可避免发生耐药。如何克服MET-TKIs耐药，首先需要了解MET 14外显子跳跃突变NSCLC对于MET-TKIs的耐药机制。由于MET 14外显子跳跃突变相对少见，且MET-TKIs临床应用时间尚短，耐药机制尚不完全清楚。研究^[63]^显示，MET-TKIs的耐药相关MET基因异常包括MET基因H1094、G1163、L1195、D1228、Y1230位点的突变以及MET 14外显子等位基因扩增。MET-TKIs继发性耐药机制包括合并TP53、KRAS、EGFR、HER3、BRAF基因扩增或突变、RAS-MAPK旁路激活、RTK-RAS-PI3K通路基因突变等^[63,64]^。依据是否存在MET基因异常，可将MET-TKIs的耐药机制分为MET依赖性耐药机制（on target）和MET非依赖性耐药机制（off target）。

目前获批用于MET 14外显子跳跃突变NSCLC的MET-TKIs均为I类MET-TKIs，通过与MET活化环中的Y1230结合，占用ATP结合袋抑制MET活性。克唑替尼作为IA类MET-TKIs，与MET主链中的氨基酸残基形成氢键，甘氨酸残基的G1163位点突变可导致克唑替尼耐药，但对IB类抑制剂敏感。D1228H/N和Y1230C/H/S突变是IB类抑制剂常见的耐药机制，这些突变对福瑞替尼^[63]^、美沙替尼^[65]^等II类MET抑制剂敏感。因此，I类MET抑制剂耐药后，可以尝试II类MET抑制剂。L1195和F1200是II类MET抑制剂耐药的常见突变位点，上述突变对IB类抑制剂可能敏感。此外，还可能存在D1228、Y1230、L1195共突变，给后续选择MET抑制剂带来困难^[63]^。因此，发生MET-TKIs耐药后，通过精准检测明确MET-TKIs的耐药机制，如明确为MET依赖性耐药机制，需根据特异性的MET基因选择后续治疗。例如，对于IA类MET-TKIs耐药的NSCLC，如合并G1163R突变，可试用IB类抑制剂；对于IB类抑制剂耐药的患者，尤其存在D1228H/N和Y1230C/H/S突变的患者，可以尝试II类MET抑制剂。

如果MET非依赖性耐药机制或更换MET抑制剂后效果欠佳的患者，如果局部或寡部位进展，也可以联合局部治疗，对于广泛转移且MET抑制剂治疗不能获益的患者，可以采用系统性的化疗或化疗联合免疫治疗或抗血管治疗等。另外，埃万妥单抗对于MET-TKIs耐药的MET 14外显子跳跃突变患者也有一定疗效。

## 总结

作为检测致癌驱动突变多基因panel的一部分，对于晚期/转移性NSCLC患者中应检测MET 14外显子跳跃突变。对于MET 14外显子跳跃突变患者，推荐（建议）一线使用MET抑制剂或作为一线治疗进展后的补救方案。随着新的MET抑制剂在我国的获批上市和临床应用，本共识将进一步推动MET标准化检测的实施，提高对MET 14外显子跳跃突变NSCLC患者规范化靶向治疗的临床实践，为中国医师的临床实践提供规范化指导。

**Competing interests**

The authors declare that they have no competing interests.

**参与本共识的专家组成员**

**执笔专家**

（以姓氏拼音首字母为序）

陈军 天津医科大学总医院

李昕 天津医科大学总医院

刘夏 天津医科大学总医院

刘东颖 天津医科大学肿瘤医院

孟庆威 哈尔滨医科大学附属肿瘤医院

邬麟 湖南省肿瘤医院

徐嵩 天津医科大学总医院

徐燕 北京协和医院

张琳琳 天津医科大学总医院

张新伟 天津医科大学肿瘤医院

赵洪林 天津医科大学总医院

**讨论专家**

（以姓氏拼音首字母为序）

陈钢 天津医科大学总医院

巩平 石河子大学医学院第三附属医院

郭占林 内蒙古医科大学附属医院

李旭 北京医院

李明江 天津市第一中心医院

李彤 天津医科大学总医院

刘京豪 天津医科大学总医院

刘明辉 天津医科大学总医院

苗茜 福建省肿瘤医院

蒲兴祥 湖南省肿瘤医院

孙大强 天津市胸科医院

徐克 天津医科大学总医院

徐医军 天津市胸科医院

杨帆 天津医科大学总医院

张鹏 天津医科大学总医院

赵青春 天津医科大学总医院
